# Intelligent Parameter Tuning Strategy for Nuclear Power Simulation Systems Based on Soft Actor–Critic

**DOI:** 10.3390/e28101125

**Published:** 2026-10-14

**Authors:** Xingyan Lu, Ze Liu, Xinyu Li, Yan Xiong, Jun Luo, Huabing Zhou

**Affiliations:** 1 China Nuclear Power Operation Technology Corporation, Ltd., Wuhan 430223, China; luxy01@cnnp.com (X.L.); xiongyan01@cnnp.com.cn (Y.X.); luojun@cnnp.com (J.L.); 2 Hubei Key Laboratory of Intelligent Robot, Wuhan Institute of Technology, Wuhan 430205, China; liuzilch@gmail.com (Z.L.); xinyuliwhu@whu.edu.cn (X.L.)

**Keywords:** nuclear power simulation system, parameter tuning, deep reinforcement learning

## Abstract

The effective tuning of extensive, high-dimensional parameter sets within nuclear power simulation systems presents considerable challenges for conventional, experience-based methodologies. Such approaches often exhibit limitations including slow convergence rates, susceptibility to local optima, and inadequate adaptability across diverse scenarios. This study introduces an automated parameter tuning strategy employing the Soft Actor–Critic (SAC) reinforcement learning approach. A SAC-based agent is formulated, where state representations, action spaces, and reward functions are specifically defined to tune parameters under predefined constraints. This facilitates the automated adjustment of complex parameter configurations. Comparative experimental evaluations indicate that the proposed SAC method reaches a solution quality comparable to that of the established baseline techniques while producing markedly more consistent results across independent runs, offering a reliable and repeatable methodology for parameter tuning in this domain.

## 1. Introduction

Growing global energy demand and the urgent need to mitigate climate change require a major transition towards sustainable, low-carbon power generation [1]. Within this context, nuclear power stands out as a key technology, offering high-capacity, dispatchable, and carbon-free electricity [2]. Its ability to provide stable baseload power is important for complementing intermittent renewable sources like solar and wind, thereby enhancing overall grid stability and energy security. However, nuclear power projects are usually large-scale and require high investment. Therefore, research into cost-effective design, operation, and maintenance strategies for nuclear power systems is needed to accelerate the clean energy transition.

Realizing the full potential of nuclear power requires rigorous standards for safety, operational efficiency, and economic viability. High-fidelity simulation systems, such as the widely used codes ATHENA [3] or RELAP [4] series, are key tools for meeting these standards throughout the lifecycle of a Nuclear Power Plant (NPP), widely used for reactor design validation, detailed safety analyses, operational strategy optimization, and personnel training [5]. The credibility of these licensing codes depends on a rigorous process of Verification, Validation, and Consistency (V&V&C) [6]. This process has evolved towards the Best-Estimate Plus Uncertainty (BEPU) framework, now a standard practice for safety assessment [7]. However, the accuracy and reliability of these simulation environments depend critically on the precise tuning of model parameters [8]. These parameters, often numerous and intricately coupled, govern the simulated behavior of complex physical phenomena within the reactor and associated systems. Therefore, accurate and efficient parameter tuning is a key requirement to ensure the reliability of the simulation results, which provide the basis for critical safety decisions and operational management. Developing tuning strategies capable of handling the inherent high-dimensionality and complexity remains a key challenge in the nuclear simulation domain.

Traditional approaches to parameter tuning in complex engineering models, while utilized, often face serious limitations when applied to modern nuclear simulation platforms [9], particularly regarding computational cost, solution quality, and adaptability [10,11]. These limitations call for developing more advanced, automated tuning strategies. Reinforcement Learning (RL), particularly Deep Reinforcement Learning (DRL), has recently emerged as an effective paradigm for addressing complex optimization problems [12], showing increasing potential within engineering systems [13,14]. Among leading algorithms, SAC [15] is known for its effectiveness in continuous control problems and sample efficiency, making it a promising candidate for the computationally intensive task of simulation parameter tuning. Its successful application in related complex nuclear power tasks further motivates its exploration here [16,17].

While RL and SAC have shown success in related nuclear control tasks, their systematic application to the specific challenge of high-dimensional parameter tuning for simulation codes remains an important research gap. This is not a trivial application, as it requires a specialized approach capable of transforming the complex tuning task into a learnable problem while ensuring the solution is both effective and reliable enough for safety-critical applications. Traditional algorithms often fall short in simultaneously meeting these demands.

To address this gap, this paper introduces a parameter tuning method based on SAC. The main contributions of this work are:

(1) An Automated and Learnable Tuning Method: We propose an automated strategy that reframes the traditional tuning workflow into a closed-loop, learnable process. This greatly accelerates the task and supports domain experts to focus on higher-level physical validation.
(2) A Novel MDP Formulation for Parameter Tuning: The core of our approach is a designed Markov Decision Process (MDP) that transforms the black-box tuning problem into a tractable reinforcement learning problem. Key contributions include a trend-informed state space for dense feedback and multiplicative updates for stability.
(3) Detailed Performance Validation: Through rigorous comparative experiments, our SAC-based approach shows improved performance compared to established heuristic methods. Most importantly, it shows stable performance, evidenced by lower standard deviations and narrower confidence intervals, providing a stable and repeatable method to establish the high-fidelity “best-estimate” models that serve as the starting point for subsequent safety analyses.

## 2. Related Work

A detailed review of nuclear engineering research indicates that high-fidelity simulation plays an essential role in the design, analysis, and safe operation of NPP [5]. While increasingly sophisticated simulation systems offer the potential for higher predictive accuracy [3], the difficulty of tuning their parameters has subsequently increased. This challenge is deeply rooted in the broader context of Code Validation and Uncertainty Quantification, which has been a central theme in nuclear safety research for decades [18]. Indeed, while intelligent and automated optimization and decision-making strategies are increasingly emerging in other industrial domains [19,20], their application to the nuanced field of nuclear parameter tuning has been sparse. Concurrently, the development of RL has facilitated its application in industrial optimization domains, garnering increasing attention over the past few decades and achieving successful applications within NPP contexts. However, the focused utilization of RL for the parameter tuning challenges intrinsic to nuclear simulation systems remains a nascent research field, with further development required. Hence, there is a pressing need to develop reliable and automated parameter tuning strategies within this demanding field.

### 2.1. Advanced Methodologies in the Nuclear Uncertainty Quantification Context

To address the challenges in parameter tuning, it is important to understand the evolution of methodologies within the nuclear field. The foundational Code Scaling, Applicability, and Uncertainty (CSAU) framework provided a structured approach to identifying uncertainty sources, including model parameters [21]. Following this, a substantial body of work has focused on developing advanced, data-driven methods for parameter estimation. This includes pioneering efforts in inverse uncertainty quantification using statistical techniques [22] and data assimilation with Bayesian inference [23]. More recently, a paradigm shift towards “intelligent methodologies” has been advocated, proposing the use of Artificial Intelligence (AI) and machine learning to create data-supported surrogate models [24] and hybrid approaches that combine simulation with deep learning [25]. Our work positions itself within this advanced, intelligent methodology trend, employing deep reinforcement learning as a new approach.

### 2.2. Traditional Parameter Tuning Algorithms and Their Limitations

NPP simulation models involve complex, coupled physical phenomena, often exhibiting pronounced non-linear behavior [26], which poses a major challenge for accurately tuning model parameters against reference data. Parameter tuning, the process of adjusting model parameters to minimize the discrepancy between simulation output and reference data, is therefore important yet computationally demanding.

Traditionally, heuristic tuning algorithms have been widely employed for such complex problems. Li et al. [9] reviewed various metaheuristic approaches, including evolutionary algorithms and swarm intelligence, for nuclear reactor fuel management optimization. Specific algorithms such as DE [27] and PSO [28] are often considered effective heuristics. However, applying these traditional methods to tune parameters in modern, high-fidelity nuclear simulation platforms frequently encounters serious limitations [10,11]. These simulations can be computationally expensive, making methods requiring numerous function evaluations prohibitive. Furthermore, the complex, often non-convex, and high-dimensional parameter landscapes can cause standard heuristics to converge prematurely to local optima or exhibit high sensitivity to hyperparameter settings. While specialized techniques like reference vector guided evolutionary algorithms [29] or dimensionality reduction combined with PSO [30] aim to mitigate some issues, the core limitations regarding computational cost and reliability in complex, expensive simulation environments often persist. Therefore, while established tools, the application of traditional heuristic tuning algorithms like DE and PSO to high-fidelity nuclear simulation tuning faces practical limitations concerning computational expense, the risk of premature convergence in complex landscapes, and sensitivity to hyperparameter settings. These challenges motivate the exploration of alternative approaches potentially offering greater speed and automation.

### 2.3. Reinforcement Learning for Complex Parameter Tuning

As an important development within AI [31], DRL has shown considerable promise for complex parameter tuning and control problems [12]. DRL’s potential has been surveyed in power systems [32], communication networks [33], and intelligent transportation systems [34]. Applications confirm its success in optimizing microgrid energy management [35], controlling thermostatically controlled loads [36], and managing building Heating, Ventilation, and Air Conditioning (HVAC) systems [37]. Within the nuclear field, DRL approaches are emerging: Multi-Layer Perception (MLP)-based RL has optimized Nuclear Steam Supply System (NSSS) supervisory control [38], DRL has addressed multi-objective optimization in NSSS [16], and DRL controllers have outperformed traditional Proportional-Integral-Derivative (PID) controllers for complex operations like plant shutdown [39]. Given the substantial computational resources and expert effort required for parameter tuning in nuclear simulations, DRL offers a potentially more automated and practical alternative, building upon these related successes.

Among advanced DRL algorithms for continuous control, SAC [15] is notable due to its effective performance and theoretical foundation. SAC operates within the maximum entropy framework, enhancing exploration and robustness by balancing expected reward maximization with policy entropy maximization. This intrinsic exploration aids in navigating the complex, potentially non-convex tuning landscapes of nuclear system simulations. Key advantages making SAC suitable for parameter tuning include its off-policy nature, enabling high sample efficiency via experience replay—important for computationally expensive simulations. Furthermore, SAC often shows good training stability and reliability, partly due to mechanisms like using double Q-networks and its capability for automatic tuning of the entropy regularization coefficient (α), reducing the manual tuning burden. Its effectiveness in continuous action spaces directly aligns with the requirements of tuning continuous-valued simulation parameters. Importantly, SAC’s suitability is supported by its demonstrated success in complex tasks within the nuclear power domain, providing strong motivation for this work. Zhang et al. [16] employed a SAC approach for multi-objective NSSS optimization. Lee et al. [40] developed autonomous operation agents using SAC-based controllers for NPP. Kim et al. [17] utilized SAC for coordinating control actions during NPP startup. Furthermore, Bae et al. [41] applied SAC with Hindsight Experience Replay (HER) for multi-objective NPP operations.

Thus, DRL, and particularly the SAC algorithm, presents a viable alternative, supported by its theoretical advantages and growing evidence of successful application to complex control and operational problems within the nuclear industry [13]. However, despite this potential, its systematic application and rigorous evaluation specifically for the parameter tuning of nuclear simulation models remains relatively underexplored. Addressing this specific gap is the primary focus of the present work. We propose and evaluate an approach using the SAC algorithm precisely for this parameter tuning challenge, aiming to show its viability and potential benefits compared to traditional methods.

## 3. Methodology

This section details the methodology developed for parameter tuning of nuclear power simulation systems, applied here to the RINSIM2 simulation platform, a modern nuclear power simulation environment developed by China Nuclear Power Operation Technology Corporation (CNPO). We first describe the overall architecture and workflow, outlining the interaction between the tuning tool and the simulation environment. Subsequently, we elaborate on the specific implementation details of the SAC algorithm and the designed MDP employed.

### 3.1. Overall Workflow and System Architecture

**Figure 1:**
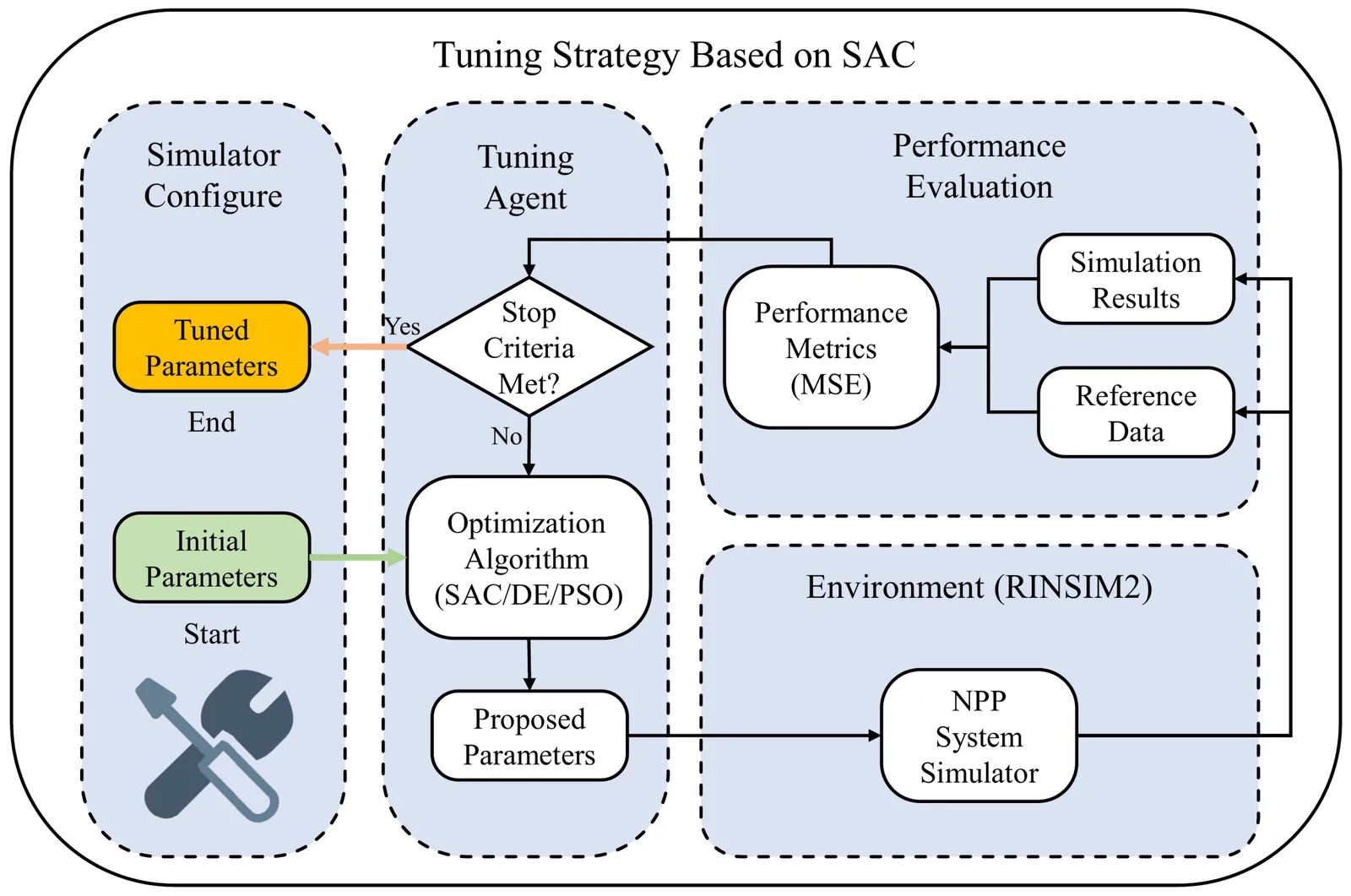
Overall architecture and workflow of the intelligent parameter tuning system interacting with the RINSIM2 simulation platform.

The overall workflow of the proposed Tuning Strategy Based on SAC is depicted in Figure 1. The process starts with Initial Parameters provided as input to the Tuning Agent. The agent’s tuning Algorithm (SAC/DE/PSO) then uses these initial parameters and subsequent performance feedback to generate Proposed Parameters. These Proposed Parameters are input to the Environment (RINSIM2), where the NPP System Simulator executes a simulation run, producing Simulation Results.

Following the simulation, the Performance Evaluation module compares the Simulation Results against predefined Reference Data to calculate Performance Metrics (Mean Squared Error, MSE). These metrics quantify the effectiveness of the Proposed Parameters for the current iteration. The calculated metrics (MSE) are then fed back to the Tuning Agent. This feedback serves two purposes: it guides the tuning Algorithm in refining its strategy to generate improved parameters for the next cycle, and it is evaluated by the stopping criteria logic. If termination conditions (e.g., MSE below a target threshold, maximum iterations reached) are not met, the loop continues. Otherwise, the loop terminates, and the final parameter set is output as the Tuned Parameters (End). This closed-loop process iteratively refines the parameters, driven by performance feedback, until a satisfactory solution is found or other stopping conditions are fulfilled.

### 3.2. SAC Framework

**Figure 2:**
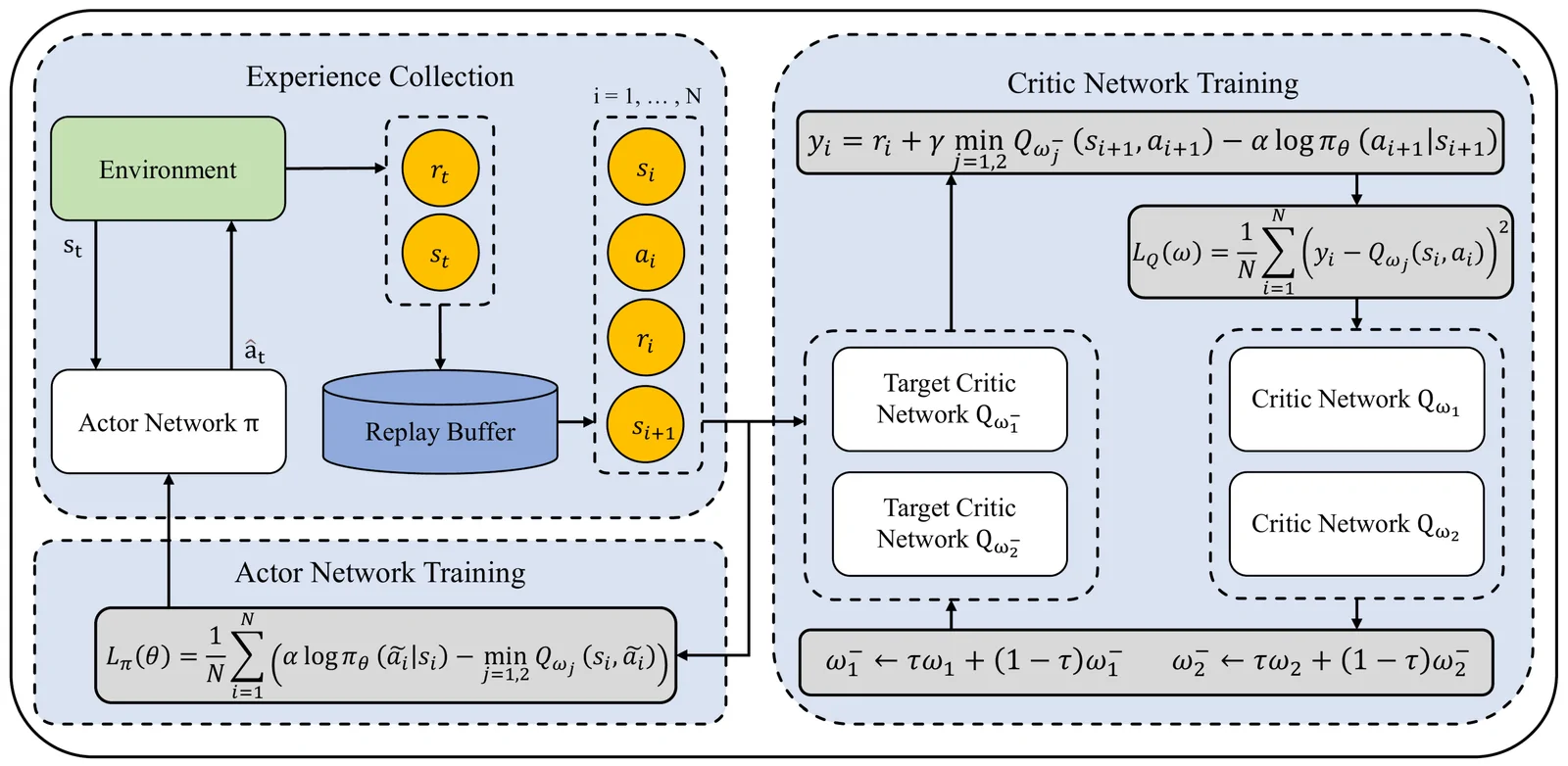
Schematic diagram of the SAC model principle.

To solve the formulated MDP, we employ the SAC algorithm (Figure 2), a prominent, off-policy algorithm well-suited for continuous action spaces. SAC’s key feature is its integration of an entropy term into the objective function, which encourages exploration and enhances training stability. The policy π is trained to maximize a trade-off between the expected return and the policy’s entropy:

(1) $$\pi^{*}=\arg\max_{\pi }E_{\pi }\left[\sum_{t}r\left(s_{t},a_{t}\right)+\alpha H\left(\pi \left(\cdot |s_{t}\right)\right)\right]$$

Here, α is the temperature parameter that balances the reward and entropy terms. The full implementation, including the updates for the actor and critic networks and the automatic entropy tuning mechanism, follows the standard approach detailed in [15]. For brevity, we omit the detailed derivations and focus on our core contribution: the MDP design.

### 3.3. MDP Formulation for Nuclear Simulator Parameter Tuning

The core contribution of this work is the MDP design for the nuclear simulation parameter tuning task. The objective is to minimize a loss function *f*, quantifying the discrepancy between simulation results ($x_{j}$) and target values ($x_{j}^{\mathrm{target}}$), potentially weighted by $\omega_{j}$ (Equation (2)), while ensuring parameter adjustments $\Delta x_{i}$ stay within predefined bounds (Equation (3)).

(2) $$f=\frac{1}{N}\sum_{j=1}^{N}{\left(\left(x_{j}-x_{j}^{\mathrm{target}}\right)\omega_{j}\right)}^{2}$$

(3) $$s.t.\;x_{i}^{\min}\leq x_{i}+\Delta x_{i}\leq x_{i}^{\max},\;i=1,2,\ldots ,P$$

To avoid ambiguity in notation, we use *N* to denote the number of evaluated output variables (N=20 in this study) and *P* to denote the number of tunable parameter coordinates; the raw 94 parameters are consolidated into P=51 physically grouped coordinates on which the agent’s actions act (Section 4.1.2). The mini-batch size used for the network updates is always stated as an explicit integer (4096) and is not denoted by a symbol. The design of each MDP component is critical for the agent’s performance and is detailed below.

#### 3.3.1. State Space ($s_{t}$)

The state $s_{t}$ observed by the agent at each step is a composite vector defined as

(4) $$s_{t}=\left\{G_{t},L_{t},\nabla L_{t}\right\}$$

The state space is designed to provide the agent with sufficient information about the current parameter settings and tuning progress. A key aspect of our proposed state representation is the inclusion of the loss trend ($\nabla L_{t}$), which gives the agent immediate, qualitative feedback on its last action’s efficacy. This lets the next action be conditioned on whether the preceding update improved or degraded the fit. The ablation study of Section 4.5 does not resolve an effect of this term on the final loss, so we attach no convergence-speed claim to it.

The full state includes the following.

Normalized Parameter Vector ($G_{t}$): This is the fundamental component, representing the agent’s current position in the high-dimensional parameter space. The original 94 parameters are grouped into 51 physically relevant sets (in RINSIM2) to reduce dimensionality and account for potential correlations. Normalization is used to keep the inputs to the neural network within a stable range.

Current Loss ($L_{t}$): The simulation model’s loss at time t (calculated using Equation (2)). This scalar value provides a direct, quantitative measure of the current solution’s quality. It answers the question: ‘How good is the current parameter set?’

Loss Trend ($\nabla L_{t}=\mathrm{sign}\left(L_{t}-L_{t-1}\right)$): This discrete signal (−1, 0, 1) provides immediate, qualitative feedback on the efficacy of the agent’s last action. It directly tells the agent if its last move was ‘Improving’, ‘Worsening’, or had ‘No Effect’.

#### 3.3.2. Action Space ($a_{t}$)

The action $a_{t}$ issued by the agent is a vector of simultaneous adjustments, one for each of the 51 grouped coordinates, defined as

(5) $$a_{t}=\left\{\Delta x_{1},\Delta x_{2},\ldots ,\Delta x_{51}\right\}\in {\left[-1,1\right]}^{51}$$

The action space represents the proposed adjustments for each of the 51 parameter groups, defined as a 51-dimensional continuous space. The key features of our proposed action space design include the use of parameter grouping to leverage domain knowledge and multiplicative updates to ensure tuning stability. This design addresses the practical challenges of dimensionality and the wide dynamic range of physical parameters.

The action space components are detailed as follows:

Parameter Grouping: The original 94 parameters were consolidated into 51 physically meaningful groups based on expert knowledge of the RINSIM2 simulator. This dimensionality reduction simplifies the learning task while also exploiting the intrinsic physical correlations between parameters, encouraging more coherent adjustments.

Multiplicative Updates: We employ a multiplicative update rule rather than an additive one. This ensures that the adjustment step ($\Delta x_{i}$) is proportional to the parameter’s current magnitude. This is important for stability, as it prevents small-valued parameters from being drastically altered while allowing for large changes to large-valued parameters.

Actions are normalized to [−1, 1] for stable training and then linearly mapped to real adjustments ($a_{t}^{\mathrm{real}}=a_{t}\cdot \beta$), where β is a scaling factor chosen to prevent excessively large parameter changes per step. The parameter groups (represented by their normalized values) are updated multiplicatively:

(6) $$G_{t+1}=G_{t}\cdot \left(1+a_{t}^{\mathrm{real}}\right)$$

#### 3.3.3. Reward Function ($r_{t}$)

The reward $r_{t}$ returned to the agent after each step combines a dense trend term and a sparser loss term, defined as

(7) $$r_{t}=\beta_{1}\cdot r_{\mathrm{trend}}+\beta_{2}\cdot r_{\mathrm{loss}}$$

(8) $$s.t.\;\beta_{1}+\beta_{2}=1$$

The reward function is structured to guide the agent effectively toward an optimal solution. The key feature of our proposed reward function lies in its hybrid structure, a common and effective strategy in reward engineering. It strategically combines a dense, local signal ($r_{\mathrm{trend}}$) for immediate, step-by-step guidance, with a global, sparser signal ($r_{\mathrm{loss}}$) to drive the agent toward the overall optimum. This balanced approach effectively mitigates the common challenge of sparse rewards in complex tuning tasks.

The reward is a weighted combination of the following.

Trend Reward ($r_{\mathrm{trend}}$): This component provides a dense and immediate reward based on the loss trend ($\nabla L_{t}$). It directly reinforces productive actions (those that decrease the loss) and penalizes counter-productive ones. By assigning a higher weight to this term ($\beta_{1}$ = 0.75), we encourage the agent to learn a stable policy that consistently moves in a promising direction.

(9) $$r_{\mathrm{trend}}=\left\{\begin{array}{cc} +1, & \nabla L_{t}=-1\;\left(\mathrm{Improving}\right)\\ 0, & \nabla L_{t}=0\;\left(\mathrm{No}\;\mathrm{Change}\right)\\ -1, & \nabla L_{t}=+1\;\left(\mathrm{Worsening}\right) \end{array}\right.$$

Loss Reward ($r_{\mathrm{loss}}$): This component reflects the global quality of the current state. It’s normalized to [−1, 1] based on estimated minimum ($L_{\min}$, e.g., 0) and maximum ($L_{\max}$, e.g., initial loss) loss values. A smaller $L_{t}$ yields a larger $r_{\mathrm{loss}}$. This prevents the agent from being satisfied with minor improvements and pushes it towards an optimal solution.

(10) $$r_{\mathrm{loss}}=2\cdot \frac{L_{\max}-L_{t}}{L_{\max}-L_{\min}}-1$$

We note that $\nabla L_{t}$ serves a dual role, appearing both in the state (Equation (4)) and in the reward (Equation (9)), and the repetition is deliberate. In the state, $\nabla L_{t}$ informs the policy about the qualitative direction of the previous transition, enabling the agent to condition its next action on whether the preceding update improved or degraded the fit. In the reward, the same signal provides an immediate, dense reinforcement of directionally correct behavior. Including a shaping-like signal in both places is a standard mechanism for mitigating sparse rewards in long-horizon optimization tasks, and the two roles are complementary rather than redundant, since the state determines what action to take while the reward determines how desirable the resulting transition was.

### 3.4. Methodological Considerations and Limitations

We acknowledge the intrinsic limitations of any parameter tuning approach when applied to complex physical models, a primary one being the issue of compensating errors. As correctly noted in the nuclear safety community, deficiencies in one physical model or correlation (e.g., turbulence modeling) may be incorrectly ‘compensated’ for by tuning the parameters of another, leading to a mathematically optimal solution that may lack physical fidelity outside the specific tuning domain.

Our proposed SAC-based approach does not claim to resolve this fundamental challenge, which stems from incomplete scientific knowledge. Instead, it functions as an effective tuning engine within the existing simulation model. The key value propositions of our approach in this context are:

Automation of the Tuning Workflow: Our approach automates the otherwise laborious process of searching for an optimal parameter set in a high-dimensional space, so that the manual effort this requires from domain experts is substantially reduced.

Robustness and Repeatability: A key advantage of our method, demonstrated by the low standard deviation in results, is its ability to consistently converge to the same optimal solution. This provides a stable and repeatable baseline for subsequent analysis, eliminating the stochasticity often associated with heuristic methods.

Ultimately, we position our approach as an effective tool to support the domain expert. The SAC agent rapidly identifies a best-fit parameter set based on the available data. The final assessment of the physical reasonableness of this parameter set, and the potential impact of compensating errors, remains a critical task for the expert engineer or physicist. Our method streamlines the first step, allowing experts to focus their efforts on the more crucial second step of physical validation.

## 4. Experiments and Results

This section details the experimental evaluation of the proposed SAC-based parameter tuning methodology compared against conventional heuristic algorithms (DE and PSO). Evaluation metrics, experimental configuration, the SAC agent training process, and comparative performance results are presented.

### 4.1. The Parameter Tuning Benchmark

#### 4.1.1. Simulation Environment and Tuning Task

The experiments are conducted using the RINSIM2 platform, a powerful simulation environment developed by CNPO. For this study, we utilize a model configured to represent a Hualong One (HPR1000) Pressurized Water Reactor (PWR) coupled with an M310 series turbine generator set. The RINSIM2 platform is a versatile tool that serves a dual role as both a high-fidelity engineering analyzer for design and safety analysis, and a full-scope simulator for operator training. This dual capability ensures that the model is both physically detailed and comprehensive in its representation of the plant’s systems. As such, it incorporates detailed physical models for thermal-hydraulics and control systems. The parameter tuning challenge addressed in this study is centered on calibrating key thermal-hydraulic parameters, predominantly the form loss coefficients (K-factors) of the secondary side fluid network. This is a fundamental challenge shared by all 1-D system codes like RELAP5, where such coefficients are used to model the pressure drop in complex components. The output variables used for performance evaluation, such as pressures, temperatures, and flows at critical locations like the moisture separator reheater and turbine sections, are likewise standard figures of merit for validating the fidelity of nuclear power plant system simulations.

The specific operational scenario selected for this tuning task is a critical phase of the NPP startup process: a power ascension from 50% to 100% Rated Power (RP). This operation is executed as a linear ramp over a two-hour period. This scenario serves as a stringent test of the model’s integrated performance, because the operating point it defines requires the accurate and coordinated response of the reactor core, control systems, and the entire thermal-hydraulic network. The performance evaluation is based on minimizing the discrepancy for N = 20 key output variables, which are predominantly located in the secondary loop. Key examples of these variables include Steam Generator Pressure, Steam Generator Level, and Main Steam Flow.

The steady state of the plant at the power-ascension operating point is the condition on which the tuning task is posed and at which the reference design data are defined. Each evaluation performed during tuning is therefore a steady-state solution of the model at that condition, not an integration of the two-hour power ascension, and the loss of Equation (11) is computed from that solution. When RINSIM2 executes a transient such as the power ascension, it advances the coupled calculation with a physical time step of 0.05 s and synchronises its constituent programs at that interval, so that the two-hour span comprises 144,000 time steps; that step is a property of transient execution and does not enter the tuning loop.

One action step of the agent is not a fixed interval of physical time either. An action step applies a single parameter update, and that update is assessed by one steady-state solution of the model at the same operating point, so that the 50 action steps of an episode are 50 successive parameter-update trials scored against the same steady-state reference rather than a partition of the two-hour span.

The wall-clock times reported in Section 4.4.1 measure the cost of re-solving the steady state after each parameter update. One evaluation therefore takes milliseconds rather than minutes, which is why the reported times bear no relation to the two-hour duration of the power ascension. The accelerated mode runs the same thermal-hydraulic model that is used for the corresponding engineering analysis, and the reported times are obtained by advancing 16 instances of that model concurrently rather than by simplifying the physical equations.

#### 4.1.2. Reference Data and Target Parameters

The objective of the tuning task is to align the simulator’s output with a “ground truth” reference dataset ($x^{\mathrm{target}}$). This dataset is a hybrid reference, primarily derived from the plant’s official design data, which specifies the expected steady-state system behaviour at the power-ascension operating point. To enhance its fidelity and practical relevance, these data were further validated and locally adjusted using key data points from actual operational records of a reference unit.

The tuning process acts on the input side of the model, adjusting 94 key parameters that predominantly characterize the thermal-hydraulic behavior of the secondary side fluid network. They were identified by domain experts as the most influential yet uncertain parameters for this operating condition, making them prime candidates for tuning. As detailed in our MDP formulation, these 94 parameters are consolidated into 51 physically correlated groups to structure the agent’s action space.

#### 4.1.3. Performance Metrics

The primary metric is the final Loss achieved by each algorithm, representing the discrepancy between the simulation output ($x_{i,j}$) and target values ($x_{j}^{\mathrm{target}}$). It is calculated as the MSE over N specific output variables, averaged across M independent tuning runs:

(11) $$\mathrm{Loss}=\frac{1}{M\cdot N}\sum_{i=1}^{M}\sum_{j=1}^{N}{\left(\left(x_{i,j}-x_{j}^{\mathrm{target}}\right)\omega_{j}\right)}^{2}$$

The inclusion of the weight factor $\omega_{j}$ is important as it directly addresses the multi-objective nature of the parameter tuning problem. In practical analyses, not all output variables are equally important. The $\omega_{j}$ term provides flexibility for domain experts to assign higher importance to critical variables, thereby guiding the tuning process to prioritize the accuracy of the most important outputs. For this foundational study and to ensure a fair comparison with the baseline heuristic methods, we have assumed equal importance for all N = 20 output variables by setting $\omega_{j}=1$ for all *j*. However, the framework is inherently capable of supporting weighted, multi-objective tuning.

To evaluate the stability and robustness of each algorithm, we computed two key statistical metrics across the M independent runs: the Standard Deviation and the 95% Confidence Interval ($CI_{\mathrm{Loss}}$, $CI_{\mathrm{time}}$) for the mean of both the final Loss and the tuning time.

The standard deviation measures the dispersion of the results, calculated as the square root of the sample variance:

(12) $$\mathrm{Std}=\sqrt{\frac{1}{M-1}\sum_{i=1}^{M}{\left(x_{i}-\bar{x}\right)}^{2}}$$

A lower standard deviation indicates greater consistency. The 95% CI provides a range within which the true mean of the performance is expected to lie with 95% probability. For a small sample size (M = 10), it is calculated using the t-distribution:

(13) $$\mathrm{CI}=\bar{x}\pm t_{\mathrm{crit}}\cdot \frac{\mathrm{Std}}{\sqrt{M}}$$

where *x* represents either the loss or time data. The term $\mathrm{Std}/\sqrt{M}$ is the standard error of the mean. $t_{\mathrm{crit}}$ is the critical value from the t-distribution for a 95% confidence level with M−1 degrees of freedom. For our study with M = 10, the degrees of freedom is 9, and the corresponding $t_{\mathrm{crit}}$ value is 2.262. Together, these metrics provide a full assessment of each algorithm’s robustness.

### 4.2. Experimental Setup

The experiments were conducted on a workstation equipped with an Intel Core i7-7700K CPU, 32 GB of RAM, and an NVIDIA GeForce GTX 1080 Ti GPU. The training and inference of the SAC agent, being based on neural networks, were accelerated on the GPU. In contrast, the population-based DE and PSO algorithms were executed on the CPU.

We performed M = 10 independent tuning runs for each algorithm. The dimensionality of the raw parameter space is 94, which is grouped into 51 for the SAC agent’s action space.

The SAC agent acts on the P=51 grouped parameter coordinates, one action corresponding to a set of simultaneous adjustments of these coordinates (Equation (5)). The DE and PSO baselines do not use this representation. Each individual is a parameter vector of the 94 raw parameters, and the population is evolved directly in that space, so that neither algorithm has a counterpart of the agent’s action. To make the comparison dimensionally matched, the baselines were additionally run in the same P=51 grouped space; both sets of results are reported in Table 1.

During the reported campaign every run of every algorithm was initialised from a single shared parameter vector, and the three algorithms share that starting point. The training of the agent is arranged separately. As described in Section 4.3.1, each training episode is initialised from one of ten distinct initial parameter sets, drawn at random for one of the parallel environments, whereas the evaluation runs reported here all start from the one shared vector. The ten evaluation runs of each algorithm are therefore independent repetitions of a single tuning task rather than ten distinct tasks, and they measure how each algorithm’s outcome varies with its own stochasticity rather than how it varies with the starting point. It is for this reason that the test described in Section 4.4.1 is an unpaired test.

A second design is used for the paired comparison. All three algorithms act on the same tuning task in the P=51 grouped space and are started from the same ten initial parameter vectors, so that the final losses obtained from a given initial vector are treated as matched. This design holds the task fixed and varies only the starting point. The comparison between algorithms on it is therefore a matched-pairs comparison, tested with the two-sided Wilcoxon signed-rank test, and the spread of the final loss across the ten initial vectors is compared with a variance-ratio *F* test.

#### 4.2.1. Baseline Heuristic Tuning Algorithm Settings

DE: We used the “DE/rand/1” strategy with a population size of 80 and a maximum of 100 iterations. The crossover probability (CR) was set to 0.5, and the mutation factor (F) was uniformly sampled from [0.1, 0.5] for each generation.

PSO: The PSO was configured with a population size of 80 and 100 maximum iterations. The cognitive and social coefficients (c1, c2) were both set to 2.05, and the inertia weight (ω) was 0.6. These settings are common in the literature and showed good performance in preliminary tests.

We acknowledge that the DE and PSO hyperparameters follow commonly used literature defaults [27,28] and were not subjected to the same per-hyperparameter tuning effort invested in the SAC agent (Section 4.3.2). This asymmetry in tuning effort is a known pitfall in RL-versus-baseline comparisons [42]; in the present setting it can only act against the baselines, in the sense that more extensively tuned heuristics might perform better than reported here. As a mitigating factor, the population sizes and iteration budgets of DE/PSO were set so that their total number of simulator evaluations (Section 4.4.1) is of the same order as the SAC training budget.

#### 4.2.2. SAC Agent Configuration

The proposed SAC agent was implemented using the Stable-Baselines3 library. The final, trained agent used for the comparative evaluation has the following configuration:

Network Architecture: Both the actor (policy) and critic (Q-value) networks are three-layer Multi-Layer Perceptrons (MLPs) with 256 neurons in each hidden layer and ReLU activation functions.

Key Hyperparameters: The configuration includes a learning rate of 0.005, Adam optimizer, replay buffer size of 4,000,000, batch size of 4096, action scaling factor (β, defined in the action space of Section 3.3.2) of 0.001, reward weights ($\beta_{1}$, $\beta_{2}$, Equation (7)) of (0.75, 0.25), a soft update coefficient (τ) of 0.005, and max epoch of 50. The rationale behind these choices is detailed in the following section.

### 4.3. Training Process and Hyperparameter Rationale

The SAC agent was trained from scratch using the Stable-Baselines3 library. To accelerate the collection of experiences, we employed a vectorized architecture with 16 parallel RINSIM2 environments running concurrently. This setup allows the agent to interact with multiple independent instances of the simulation, significantly improving data collection efficiency. The training process and key hyperparameter choices are detailed below.

#### 4.3.1. Training Workflow

**Figure 3:**
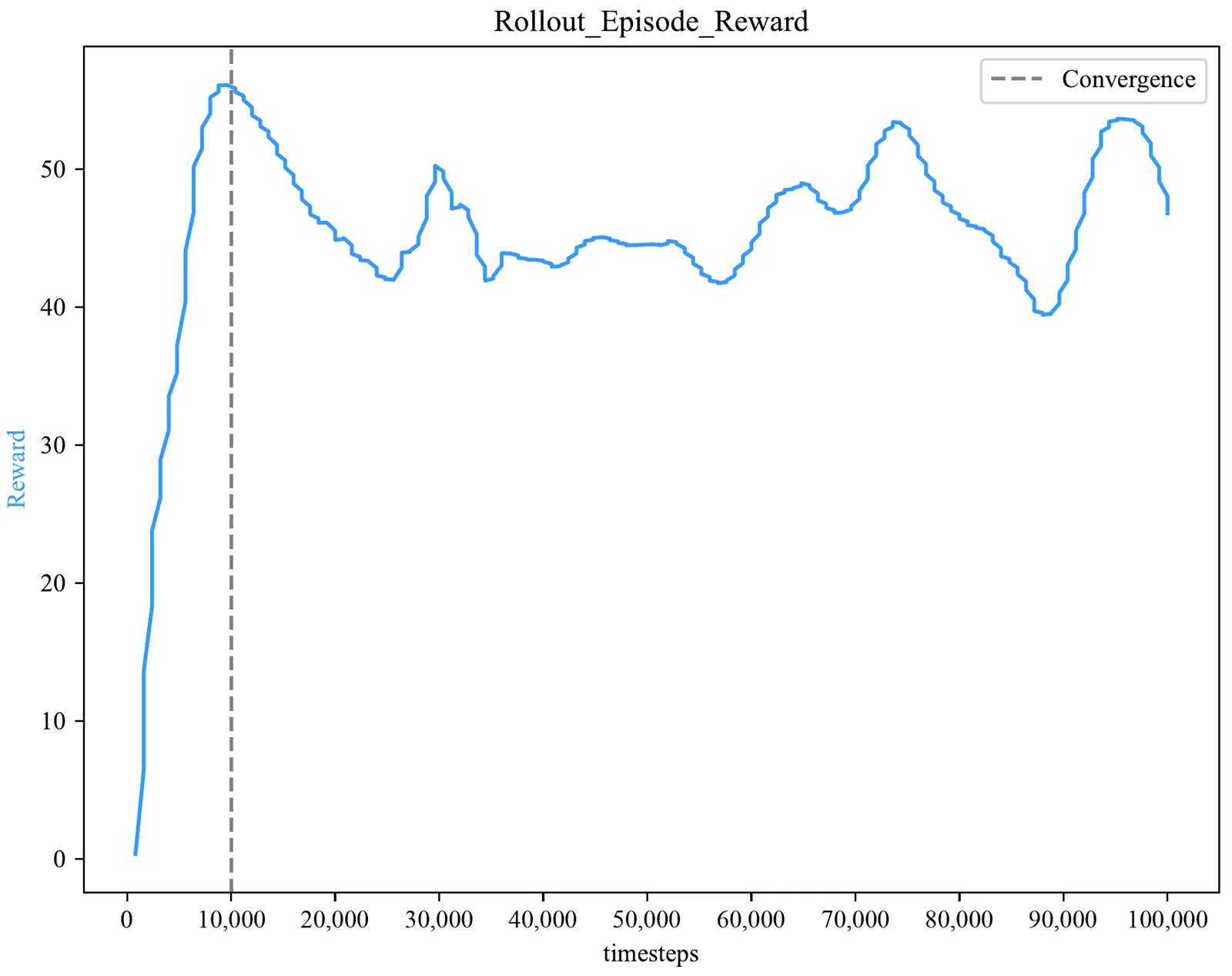
Evolution of the agent’s episode reward during training, with convergence marked at timestep 10,000.

**Figure 4:**
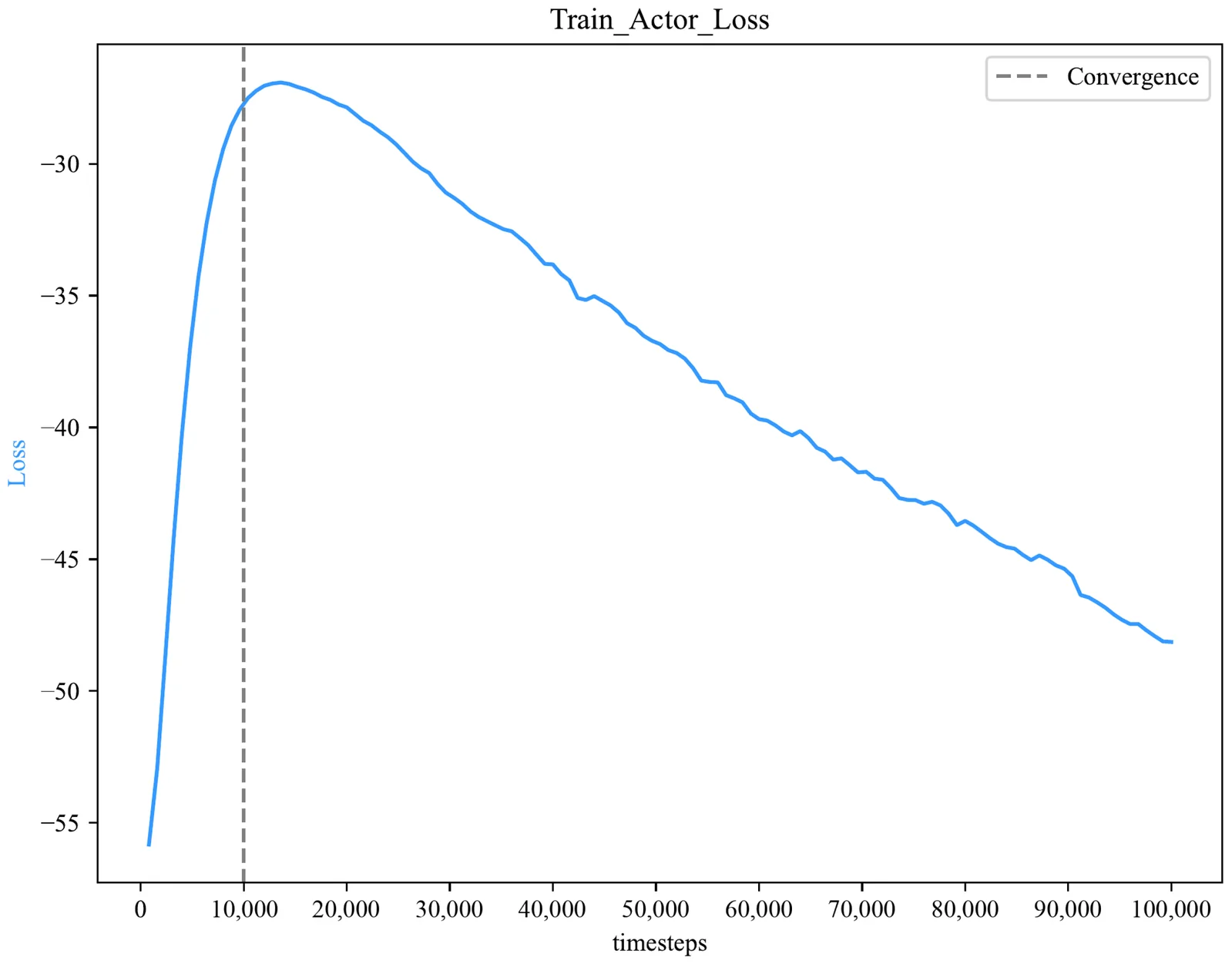
Actor network loss during training, with convergence marked at timestep 10,000.

**Figure 5:**
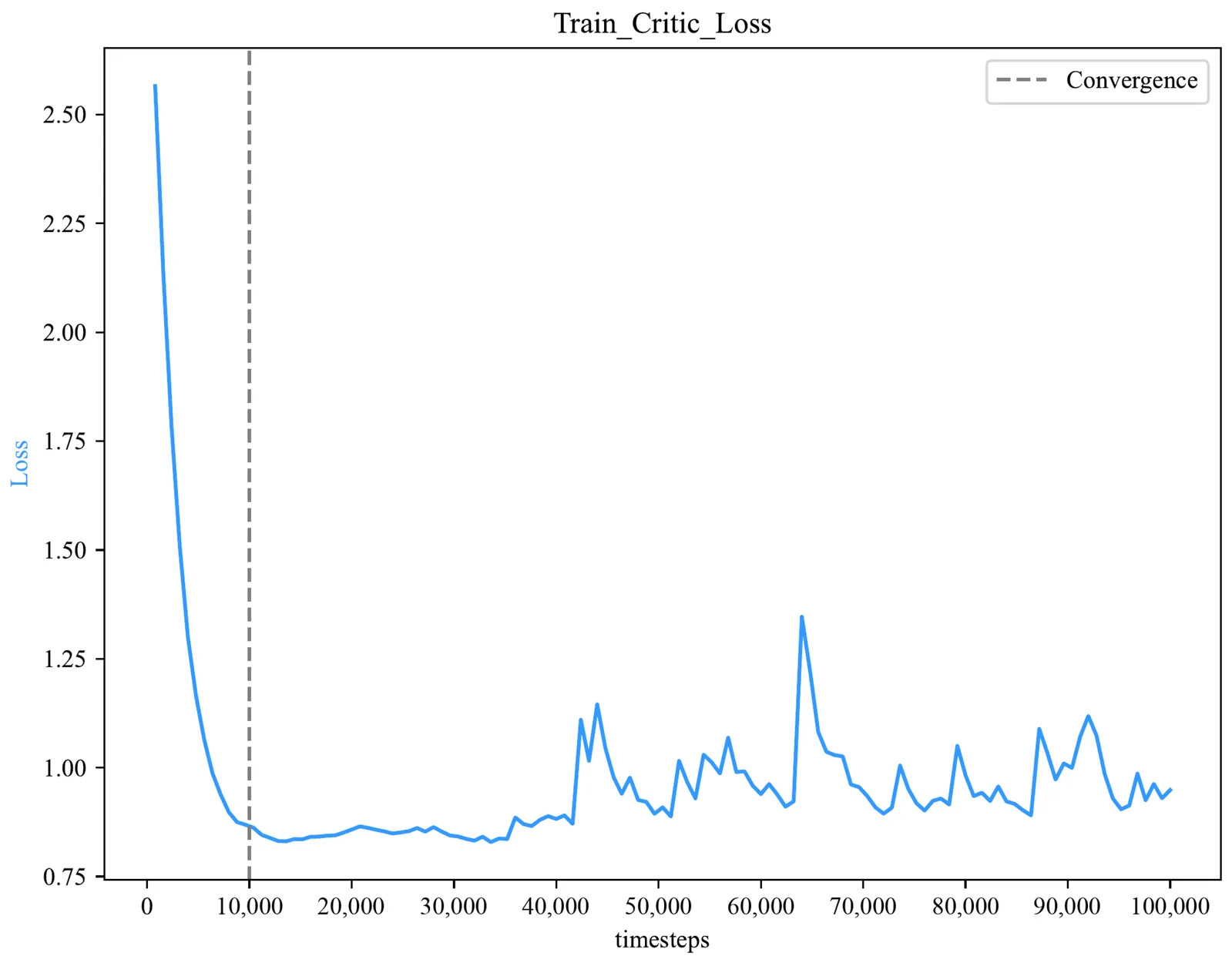
Critic network loss during training, with convergence marked at timestep 10,000.

**Figure 6:**
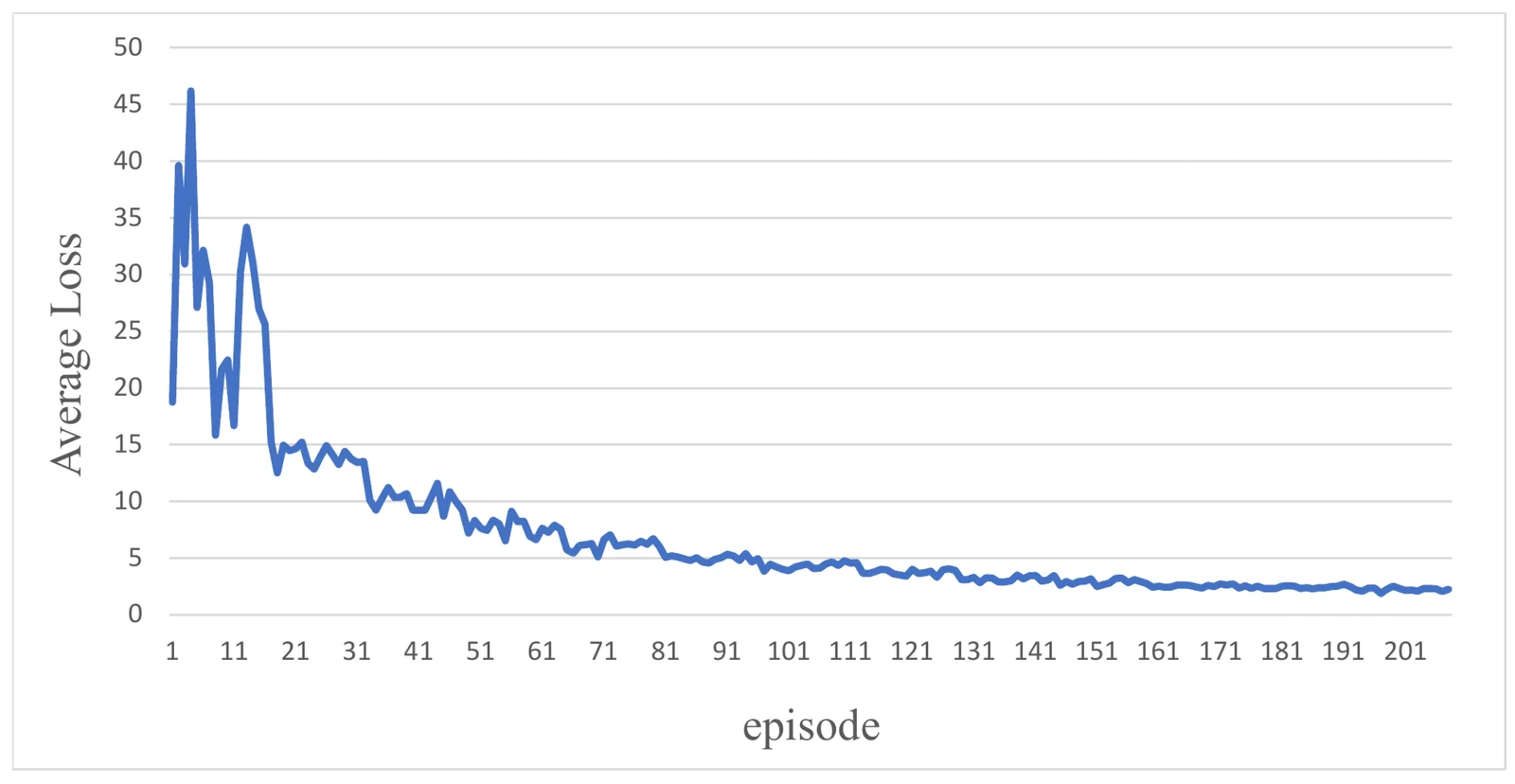
Evolution of the Loss metric during agent training.

The training loop proceeds as follows: At the beginning of each episode, one of the M = 10 distinct initial parameter sets is randomly selected to initialize one of the parallel environments. The agent then interacts with the environment for a fixed number of steps, defined as Max Epoch = 50. The transition tuples ($s_{t},a_{t},r_{t},s_{t+1}$) collected from all 16 environments are stored in a unified Replay Buffer. The agent’s networks are updated by sampling mini-batches from this buffer. The convergence during training is illustrated by the trajectories of the reward and network losses (Figure 3, Figure 4 and Figure 5). The agent typically reached convergence within approximately 10,000 training steps. The total training duration was set to 10,400 steps to ensure all parallel episodes completed fully (calculated based on Max Epoch and number of environments). The evolution of the average primary Loss metric (Equation (11)) across the 10 independent runs, depicted in Figure 6, confirms effective learning and tuning via the designed MDP.

#### 4.3.2. Hyperparameter Selection and Rationale

The choice of hyperparameters is critical for the stability and performance of the DRL agent. Our settings, listed in Section 4.2, deviate from the standard single-environment defaults to effectively leverage our vectorized training architecture with 16 parallel environments. The rationale for these adjustments is as follows:

Replay Buffer Size (4,000,000): A large buffer is important for off-policy algorithms like SAC to break temporal correlations in the data. With 16 environments generating experiences simultaneously, a larger buffer is necessary to maintain a diverse and representative history of transitions from many different trajectories, preventing the agent from overfitting to recent, potentially correlated, data from a few specific episodes. We scaled the default buffer size to accommodate this increased influx of diverse data.

Batch Size (4096): We substantially increased the batch size from the typical default (e.g., 256). This is a direct consequence of using multiple parallel environments. A larger number of parallel workers increases the variance of the collected data per update step. To obtain a stable and reliable gradient estimate from this more varied data, a larger mini-batch is required. This ensures that each training step averages over a wide range of experiences, smoothing the learning process and preventing unstable updates.

Learning Rate (0.005): In large-batch training regimes, it is common practice to use a larger learning rate. According to principles like the “Linear Scaling Rule” [43], a larger batch size reduces the noise in the gradient, allowing for more aggressive update steps without destabilizing the training. We found that a learning rate of 0.005, paired with our large batch size, led to faster and more stable convergence compared to the smaller default learning rate.

Action Scaling Factor (β = 0.001): This small scaling factor is a critical design choice for ensuring training stability. It constrains the magnitude of the parameter updates at each step, preventing drastic changes that could either lead to numerically unstable simulations in RINSIM2 or cause the learning process itself to diverge.

Reward Weights ($\beta_{1}$, $\beta_{2}$): A higher weight was assigned to the trend-based reward ($r_{\mathrm{trend}}$). This prioritizes teaching the agent to make directionally correct moves (i.e., actions that lower the loss), providing a dense and consistent learning signal that guides the agent effectively, especially in the early stages of training.

Soft Update Coefficient (τ = 0.005): A small value for τ ensures that the target networks update slowly. This practice is standard in DRL and provides a stable target for the critic and actor updates, preventing moving-target problems and improving overall learning stability.

### 4.4. Results and Discussion

#### 4.4.1. Result

Three distinct sets of runs underlie the results reported below, and to prevent conflating them we state at the outset which table reports which: the ten runs of the submitted campaign form the SAC row of Table 1; the matched re-run of the two baselines in the P=51 grouped coordinates forms its two rows marked *grouped*; and the paired design, in which the three algorithms act on the same task from the same ten initial parameter vectors, is reported in Table 2. The two remaining rows of Table 1 repeat the original P=94 results of the submitted version, with no new runs behind them.

To evaluate the performance of the proposed tuning strategy, we first established a baseline case using a set of pre-existing, un-tuned model parameters. This baseline represents a typical starting point in an engineering tuning task, yielding a high initial loss of 111.29. The objective for all tested algorithms was to minimize this loss by automatically adjusting the parameters to better match the reference data.

After training, the SAC agent was evaluated in deterministic mode (entropy coefficient α = 0). For PSO and DE, the global best solution $G_{\mathrm{best}}$ and its corresponding metrics were recorded after the maximum iterations. Table 1 presents the performance metrics averaged over the 10 independent tuning runs.

Table 1 collects the results. Its three upper rows were obtained in the P=51 grouped space. The SAC row is from the campaign reported in the submitted version, and the two rows marked *grouped* are from the additional runs of the baselines in that same space, on a different set of ten tasks. The two sets therefore come from separate campaigns and are not a single paired experiment. The last two rows repeat the original P=94 results of the submitted Table 1, so that the two spaces can be read one against the other. Every evaluation counted in the *Sim. calls* column is a steady-state solution of the model at the operating point being tuned, with DE and PSO performing 80×100=8000 evaluations and SAC 10,400 environment steps.

One entry in the upper block needs a word of explanation. DE reaches 29.85±9.64 in the grouped coordinates, well above its 1.72±0.94 in the raw space, whereas PSO is barely affected by the change in space (3.27±1.54 against 2.21±1.33); both baselines ran the same ten tasks in the grouped coordinates, and DE was the worse of the two on all ten, so the gap belongs to DE’s own search there rather than to the space itself.

In terms of tuning accuracy, all three algorithms reduced the initial loss from 111.29 to mean values between 1.65 and 2.21, i.e., by more than 98% relative to the un-tuned baseline, confirming that the tuning task is tractable for all tested methods. The SAC agent achieved the lowest mean final loss (1.65), followed by DE (1.72) and PSO (2.21). We note that the mean improvement of SAC over DE is modest (4.1%) and that the 95% confidence intervals of their mean losses overlap considerably ([1.53, 1.78] versus [1.05, 2.40]). The advantage of SAC visible in Table 1 is therefore not primarily a mean-accuracy gain but a consistency gain: its run-to-run dispersion is markedly smaller, as quantified in the statistical analysis below.

On tuning efficiency, two measures have to be kept apart. The primary one in this benchmark is the number of RINSIM2 evaluations (simulator calls), because that count is the same for a given method whatever hardware it runs on and however many instances are advanced in parallel. DE and PSO performed 80×100=8000 evaluations in total, whereas SAC interacted with the simulator for 10,400 environment steps, each step corresponding to one evaluation of the steady state at the tuned operating point (Section 4.3). SAC thus consumed somewhat more simulator calls than the baselines, which agrees with the reviewer’s observation. All three lie within the same order of magnitude (≈$10^{4}$), and we make no efficiency claim on this measure.

For completeness, the average wall-clock runtime was 39.14 s for SAC versus 61.84 s for DE and 72.29 s for PSO. SAC training, however, used GPU acceleration with 16 parallel RINSIM2 environments, whereas DE and PSO were executed sequentially on the CPU (Section 4.2). The wall-clock runtimes are therefore not a hardware-matched comparison of algorithmic efficiency, and we state this asymmetry as a limitation of the present study.

The reported SAC runtime covers the full training process (10,400 steps). Once the policy is trained, applying it to a new case costs less than one second.

Each of the 10,400 training steps corresponds to one RINSIM2 evaluation, and Section 4.1.1 sets out what that evaluation is. The reported runtimes measure the cost of perturbing the parameter set and re-solving the steady state, which is a millisecond-scale operation in the accelerated simulation mode on the workstation hardware described in Section 4.2. It is this capability that makes the large number of function evaluations required by the learning process and by the population-based baselines computationally tractable.

Finally, a notable difference was observed in the stability of the results. The standard deviation of the final loss for the SAC agent (0.17) was substantially lower than that of DE (0.94) and PSO (1.33). Similarly, the 95% confidence interval for the SAC’s mean loss, [1.53, 1.78], was narrower than the intervals for DE [1.05, 2.40] and PSO [1.26, 3.16], indicating a higher degree of run-to-run consistency for the proposed method.

To support the robustness comparison beyond the descriptive statistics of Table 1, we applied a two-sided Mann–Whitney *U* test to the final loss and a variance-ratio *F* test to the run-to-run standard deviations. All runs of the campaign started from one common initial parameter vector, so the ten runs of an algorithm are independent repetitions from that point rather than ten matched pairs across algorithms, and the unpaired *U* test is the appropriate choice. The per-run losses of PSO from that campaign are no longer recoverable, so the tests are reported for SAC against DE only, and the descriptive statistics of PSO in Table 1 are left to stand on their own.

On the mean the *U* test gives p=0.141 with a Cliff’s delta of 0.40, which does not reach significance at the α=0.05 level. On the run-to-run dispersion the variance-ratio *F* test gives $p=2.7\times 10^{-5}$ with a ratio of standard deviations of 0.19.

The practical advantage of the proposed method is not primarily the mean accuracy margin but the markedly lower run-to-run dispersion (Std of 0.17 versus 0.94 for DE) within that same campaign, and the *F* test supports that.

On the paired design described in Section 4.2, where the three algorithms act on the same task and share the same ten initial parameter vectors, the two-sided Wilcoxon signed-rank test on the final loss gives p=0.0020 against DE and p=0.2324 against PSO, and the variance-ratio *F* test on the spread of the final loss across the ten initial vectors gives $p=4.8\times 10^{-11}$ against DE and $p=1.2\times 10^{-8}$ against PSO. Table 2 gives the outcome.

In that table every statistic compares the proposed method against the baseline named in the row. The *SAC better* column counts the initial vectors on which the proposed method attains the lower final loss, the Wilcoxon column gives the *p*-value of the two-sided signed-rank test over the ten matched pairs, and the last column gives the *p*-value of the variance-ratio *F* test on the run-to-run variances, proposed method over baseline. The variance ratios themselves are 0.0017 against DE and 0.0059 against PSO.

To place the aggregated loss reduction in an engineering context, Table 3 reports the deviations of representative output variables in physical units, taken from the ten runs of the agent on the tuned operating condition and from the shared untuned initial parameter set used as the baseline. Each post-tuning value is the mean over the ten runs of the submitted campaign, taken from the returned parameter set of that run; pressures are reported in Pa and the flow variables in kg/s. After tuning, the deviation of the low-pressure steam pressure falls from 42.75 to 0.556 Pa, that of the main steam flow from 1.168 to 0.265 kg/s, that of the MSR flow of the second train from 1.481 to 0.061 kg/s, and that of the MSR flow of the first train from 0.430 to 0.067 kg/s.

The reduction in the physical deviations is of the same order as the reduction in the aggregated loss, and it is concentrated in the variables whose reference deviations were physically significant at the tuned condition. The variables not listed carry reference deviations two to five orders of magnitude smaller, and their absolute deviations stay below the resolution at which the corresponding measurements are reported.

#### 4.4.2. Discussion

The results indicate that the proposed SAC-based approach attains a final loss no worse than that of the heuristic baselines while producing a run-to-run dispersion markedly smaller than that of DE. In safety-critical applications such as nuclear simulation, a tuning method that reliably converges to a solution of comparable quality with small run-to-run variance (Std of 0.17 versus 0.94 for DE) is more valuable than a method that occasionally finds a comparable optimum but exhibits much larger dispersion. The exploration strategy guided by our designed MDP navigates the grouped 51-dimensional parameter space consistently across independent runs, whereas the stochastic search patterns of the heuristic methods remain more sensitive to their random initialization. In particular, the narrower confidence intervals indicate a higher degree of run-to-run consistency and predictability for the proposed method, which is a key advantage in safety-critical applications where repeatable outcomes are essential. From an efficiency standpoint, all methods required a comparable number of simulator calls; the practical advantage of the trained SAC agent is that, once trained, the learned policy can be re-applied to new tuning cases at negligible inference cost, whereas DE and PSO must restart their population-based search from scratch.

The practical implications of these findings are noteworthy. By automating and accelerating the parameter tuning workflow, this approach frees domain experts to focus on higher-level analysis. This enhanced efficiency and confirmed reliability in establishing a “best-estimate” model improve the quality of the starting point for subsequent safety analysis and operator training, although the physical plausibility of the tuned parameter set must still be confirmed by expert judgement. Looking further, such a fast and automated tuning capability is a key step towards building more adaptive and responsive simulation environments, which are important for modern nuclear plant operation and lifecycle management.

However, several limitations define the scope for future work. The performance of the tuned parameter set with noisy experimental data has not been characterized. The current MDP design is also designed for the RINSIM2 platform, and its transferability to other system codes like RELAP5 requires further research. Despite these limitations, the methodology shows considerable potential for scalability, having proven effective in a high-dimensional action space with a scalable training architecture. These characteristics, combined with the clear path for future validation, underscore the long-term viability of this approach. For transparency, we further note that several of the authors are affiliated with CNPO, the developer and owner of the proprietary RINSIM2 platform used as the benchmark in this study. The evaluation therefore reflects an in-house industrial calibration task, and the reported comparisons should be interpreted with this context in mind.

The parameter set identified by the agent was calibrated against the steady state of a single operating condition, that of the power ascension from 50% to 100% of rated power. Its transferability to other operating points and to transients, including SCRAM, has not been tested here, and the parameter set should therefore not be assumed to remain viable outside the condition for which it was identified. Extending the validation to other transients is left to future work, and in the interim the scope of the reported results is the tuned operating condition alone.

### 4.5. Ablation Study

To verify which of the two design choices highlighted as contributions the measurements actually support, we conducted an ablation study. All four configurations were run from the identical initial parameter vector, and the runs of the four configurations are matched one to one; the comparison is therefore made with a two-sided Wilcoxon signed-rank test over the ten runs, applied to each variant against the full model. Table 4 collects the measurements and the significance of each difference.

The multiplicative form of the update is confirmed to matter. Replacing it with an additive update raises the final loss from 1.67±0.17 to 13.72±12.50 (p=0.002). The trend term in the reward is confirmed as well. Removing it raises the loss to 4.90±1.52 (p=0.002).

The trend-informed state is the one component this experiment does not resolve. Removing $\nabla L_{t}$ raises the loss only to 1.87±0.23, a change of 12.5% that does not reach significance under the signed-rank test (p=0.193). We have narrowed that claim alone. The trend-informed state is presented as a reasonable design element of the MDP formulation rather than as a contribution this experiment confirms, while the claims attached to the multiplicative update and to the dense reward shaping now rest on the measurements above.

## 5. Conclusions

This study proposed an automated parameter tuning strategy for nuclear power simulation systems, using the SAC reinforcement learning algorithm. Our work is motivated by the critical need for reliable and effective simulation tools to support the BEPU framework, a cornerstone of modern nuclear safety analysis. The primary contribution of this research is not merely the application of an advanced AI algorithm, but the development of a designed approach that addresses the practical challenges of parameter tuning in the engineering domain.

Our key findings confirm the effective tuning performance of the proposed SAC-based approach. Starting from a realistic, un-tuned baseline case with high initial error, our approach reduced this prediction error by over 98%, while matching the final accuracy of the traditional heuristic methods at a markedly lower run-to-run dispersion. Importantly, it exhibits a high degree of robustness and repeatability. This is a key advantage, as it provides a stable and consistent “best-estimate” baseline for the subsequent safety analyses required in the BEPU framework.

Methodologically, we acknowledge the intrinsic challenges of this domain. We have clarified that our method is equipped to handle the multi-objective nature of tuning through a weighted loss function, allowing experts to prioritize safety-significant parameters. We also position our tool as a means to support, not replace, domain experts in confronting the core issue of compensating errors. By automating the high-dimensional search, our method allows experts to focus on the key task of validating the physical reasonableness of the optimal parameter set identified.

In addition, the direct output of our methodology is a tuned, “best-estimate” parameter set, which reproduces the reference data far more closely than the un-tuned starting point. Once the physical plausibility of that parameter set has been confirmed by expert judgement, it can serve as a starting point for a full BEPU analysis of plant transients. A complete uncertainty quantification of the kind required for licensing purposes could then be performed, outside the scope of the present work, by applying established non-parametric statistical methods such as order statistics. Our method provides a reliable means to establish the foundational best-estimate case, thereby streamlining the entire BEPU workflow.

Looking forward, the most critical next step is the rigorous validation of the methodology to show its broader applicability. Therefore, a key focus of our future work will be to apply our SAC-based approach to a standard system code, such as RELAP5, and benchmark its performance against experimental data from integral test facilities (ITFs). This will serve to validate the transferability of our approach to different simulation platforms and fulfill the gold standard of code validation in the nuclear community. Overall, this work shows DRL as an effective methodology for creating more efficient, reliable, and verifiable automated parameter tuning tools, thereby streamlining a critical component of the nuclear simulation workflow that underpins safety analysis.

## Figures and Tables

**Table 1:** Performance metrics of the tuning algorithms. *grouped* denotes the matched P=51 space and *raw* the original P=94 space.

Algorithm	Loss (±Std)	Time (s) (±Std)	95% CI (Loss)	95% CI (Time)	Sim. Calls
SAC (GPU, 16 env.)	1.65±0.17	39.14±3.03	[1.53, 1.78]	[36.97, 41.30]	10,400
DE (grouped)	29.85±9.64	18.25±0.07	[22.95, 36.74]	[18.20, 18.30]	8000
PSO (grouped)	3.27±1.54	18.34±0.45	[2.17, 4.37]	[18.02, 18.67]	8000
DE (raw)	1.72±0.94	61.84±12.91	[1.05, 2.40]	[52.61, 71.09]	8000
PSO (raw)	2.21±1.33	72.29±11.33	[1.26, 3.16]	[64.18, 80.39]	8000

**Table 2:** Paired comparison of the three algorithms on the same tuning task, from the same ten initial parameter vectors.

Baseline	Loss ± Std	SAC Better (of 10)	Wilcoxon *p*	*F* Test *p*
DE	105.70±90.59	10	0.0020	$4.8\times 10^{-11}$
PSO	30.13±48.92	6	0.2324	$1.2\times 10^{-8}$
SAC (proposed)	7.82±3.76	—	—	—

**Table 3:** Physical-unit deviations of representative output variables before and after tuning.

Output Variable	Unit	Pre-Tuning	Post-Tuning	Reduction
steam_lpo_p (LP steam pressure)	Pa	42.75	0.556	98.7%
steam_main_f (main steam flow)	kg/s	1.168	0.265	77.3%
extra_msr2_f (MSR flow, train 2)	kg/s	1.481	0.061	95.9%
extra_msr1_f (MSR flow, train 1)	kg/s	0.430	0.067	84.5%

**Table 4:** Ablation study of the MDP design choices. Each entry is the mean ± Std of the final loss of the tuned parameter set over M=10 runs, and Δ and *p* are measured against the full model.

Variant	Loss (±Std)	Δ vs. Full (%)	*p* vs. Full
Full model (proposed)	1.67±0.17	—	—
A (additive updates)	13.72±12.50	+724	0.002
B (state without $\nabla L_{t}$)	1.87±0.23	+12.5	0.193
C (reward without $r_{\mathrm{trend}}$)	4.90±1.52	+194	0.002

## Data Availability

The RINSIM2 simulator (version 2.0) used in this study is proprietary software owned by China Nuclear Power Operation Technology Corporation (CNPO) and cannot be publicly released. The complete MDP formulation, network architecture, hyperparameter configurations, and the settings of the baseline algorithms (DE and PSO) are fully described in the manuscript. Further implementation details, including the per-run results underlying the statistical tests, are available from the corresponding author upon reasonable request.
